# The Hydrolysis of Pigment-Protein Phycoerythrin by Bromelain Enhances the Color Stability

**DOI:** 10.3390/foods12132574

**Published:** 2023-06-30

**Authors:** Yifei Sun, Yuanmeng Cui, Ruhua Wang, Junrui Ma, Haili Sun, Lei Cheng, Rui Yang

**Affiliations:** 1 State Key Laboratory of Food Nutrition and Safety, College of Food Science and Technology, Tianjin University of Science & Technology, Tianjin 300457, China; 2 Beijing Engineering and Technology Research Center of Food Additives, Beijing Technology & Business University (BTBU), Beijing 100048, China

**Keywords:** phycobiliprotein, enzymatic hydrolysis, peptide, phycoerythrobilin

## Abstract

Phycoerythrin (PE) is a natural protein–pigment complex with a strong pink color, but it is sensitive to thermal and light variations. In this study, PE was extracted from *Porphyra haitanensis* in a yield of 0.2% (*w*/*w*). The phycoerythrin hydrolysates (PEH) (3–10 kDa) were prepared by enzymatic hydrolysis of PE with bromelain (8000 U/g) at 47 °C for 30 min, with a degree of hydrolysis (DH) of 11.57 ± 0.39% and a color degradation rate of 7.98 ± 0.39%. The physicochemical properties of PEH were evaluated. The UV and fluorescence spectra indicated that bromelain changed the microenvironment around phycoerythrobilin (PEB). The infrared spectrum revealed that the bromelain hydrolysis increased the *α*-helix content of PEH. The scanning electron microscope showed that bromelain destroyed the dense and smooth structure of PE, resulting in irregular porous structures. The radical scavenging activities of DPPH and ABTS of PEH were increased relative to that of PE (*p* < 0.05). The thermal (50–80 °C)-, UV (0.5–3 h)-, visible light irradiation (2–8 h)-, and metal ion exposing stabilities of PEH were significantly improved (*p* < 0.05). This study provides a potential scheme for overcoming the sensitivity of PE to thermal and light variations and facilitates PEH as a natural colorant ingredient in food and pigment applications.

## 1. Introduction

**Figure 1:**
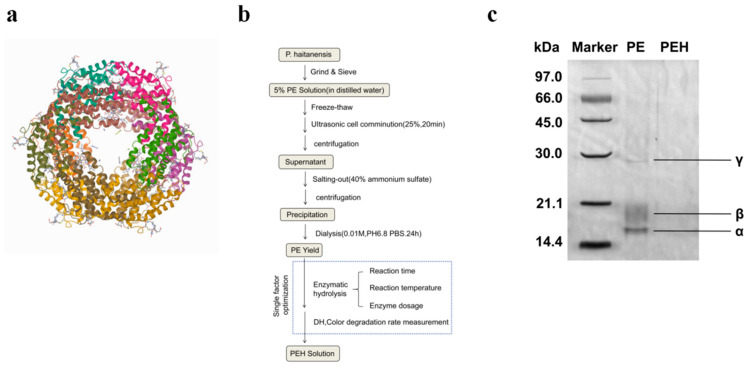
(**a**) Ribbon crystal structure of PE (PDB code: 1b8d). (**b**) Summary protocol for the extraction of PE from *P. haitanensis* and enzymatic digestion with bromelain. (**c**) SDS-PAGE analysis of PE and PEH (lane marker represents the corresponding molecular mass markers).

Phycobiliprotein is a proteinaceous natural product from algae with great application potential [1]. Phycobiliprotein participates in algal photosynthesis by assisting light harvesting and can be widely used as natural pigments in food, cosmetics, dyes, and chemical industries. Phycobiliproteins are mainly divided into allophycocyanin (APC), phycocyanin (PC), and phycoerythrin (PE) [2], of which PE is a water-soluble protein with strong red, pink, or purple color. The PE molecule is composed of apoprotein (Figure 1a) and several pigments with tetrapyrrole as the basic structure. The protein part is a class of oligomeric proteins, and the representative phycobilins are phycoerythrobilin (PEB), phycourobilin (PUB), and phycocyanin (PCB), where PEB is a natural pigment with potential applications in the food industry, such as bubble gum, beverages, milky products, and jelly. PE has a *γ*-subunit located in the central cavity; it can rivet two face-to-face (*αβ*)_3_ three aggregates and exist in various aggregated states, such as dimer, trimer (*αβ*)_3_, and hexamer (*αβ*)_6_ forms [3], and the interactions between the *α* and *β* subunits are very strong and require extreme pH and urea concentration to be dissociated [4]. The main forces to stabilize the PE are hydrophobic interaction, surface complementarity, Van der Waals interaction, and electrostatic interaction.

In practical applications, PE has disadvantages in food and dye applications due to its low stability [5]. For example, the thermal sensitivity of PE can cause the color of bake-dried Nori flakes to change from red to green [6]. A pressure of 110–250 MPa can cause PE hexamer dissociation and even protein dissociation, influencing conformational changes in the protein tetrapyrrole chromophore without affecting R-PE structure and stability [7]. Enzymatic hydrolysis is a mild and efficient approach for food processing. The relevant study indicates that a purity of 1.09 (80% yield) of APC extraction could be achieved by surfactant and enzyme-assisted extraction method [8]. The extraction rate of PE increased by 62 times using xylanase compared to that without enzyme treatment [9]. However, there are few reports on the use of enzymatic hydrolysis to study the biological activities of hydrolyzed products of PE. As a cysteine protease, bromelain is a commercial enzyme widely used in the food industry, and it is a safe and environmentally friendly protease for protein hydrolysis. The bromelain enzymatic hydrolysis has a significant effect on the improvement of functional properties and biological activities of proteins. For example, bromelain can prevent protein precipitation after bottling [10], and it can also enhance the water absorption capacity and oil binding capacity of soy protein [11]. The product of bromelain hydrolysis of pig liver has an antioxidant activity [12]. However, there is currently a lack of research on the effect of bromelain on pigment stability. At present, the pigments commonly used in the market are curcumin, carotene, etc. This research is intended to enrich the types of pigments in the market to better meet the market demand for natural pigments and also provides a potential solution for overcoming the instability problem of natural pigments.

In order to elucidate the effect of bromelain on the stability of PE, the effect of enzymatic hydrolysis on the structure and physicochemical property of PE was considered first. Additionally, PE was hydrolyzed by bromelain to obtain PEH, and the optimized hydrolysis condition was achieved by detecting the degree of hydrolysis (DH) and color degradation rate. UV/Vis and fluorescence were used to study the changes of PEH, Fourier transform infrared spectrum was used to study the changes of protein structure, and a scanning electron microscope was used to study the changes of appearance and morphology. Moreover, the antioxidant activity and the thermal, light, and metal ion exposing (Fe^3+^, Cu^2+^, Mn^2+^, Mg^2+^ and Zn^2+^) stabilities of the PE and PEH were evaluated to provide valuable information for the PE applications.

## 2. Materials and Methods

### 2.1. Materials

Bromelain (enzyme activity of 300 U/mg) was purchased from Shanghai McLean Biochemical Technology Co., Ltd. (Shanghai, China) *P. haitanensis* was purchased from Shandong Qiangsheng Food Development Co., Ltd. (Tengzhou, China). DPPH (1,1-Diphenyl-2-Picrylhydrazine) was purchased from Tixiai (Shanghai) Chemical Industry Development Co., Ltd. (Shanghai, China). ABTS (2, 2′-azino-bis(3-ethylbenzothiazoline-6-sulfonic acid)) free radical scavenging ability detection kit (BC4770) was purchased from Beijing Soleibo Technology Co., Ltd. (Beijing, China). All other chemicals were of analytical grade and purchased from Sinopharmaceutics Tianjin Co., Ltd. (Tianjin, China).

### 2.2. Preparation of PE

PE from *P. haitanensis* was extracted at a low temperature, according to the previously reported repeated freeze-thaw method [13]. The purchased dried seaweed was grinded and was sieved through a 100-mesh sieve to form a finer dry powder, followed by soaking the powder (3 g) in distilled water (60 mL). The mixture was stirred for about 5 min until uniform and was frozen and thawed for 3 times (freeze and thaw at −20 °C and 4 °C). The freeze-thaw solution was taken out, stirred with a magnetic stirrer for 3 h, and was diluted with distilled water to 150 mL, followed by sonication (125 W, 20 min, 4 °C) and filtering with gauze. The obtained liquid was centrifuged for 20 min (10,000 r/min, 4 °C), and the supernatant was collected as the crude PE extract. Ammonium sulfate was added to the crude extract with 40% saturation, and the mixture was centrifuged for 20 min (10,000 r/min, 4 °C). The precipitate was dissolved in the distilled water, and the PE precipitate was further dialyzed with phosphate buffer (0.01 M, pH 6.8) for 24 h to obtain a PE extract. The yield of PE was calculated using the following (Formula (1)) [14]:

Yield (%) = (PC × V)/DB(1)

where yield is the yield of the extracted PE (PE/mg of dried biomass), PC is the concentration of PE (mg/mL), V is the volume of solvent (mL), and DB is the dried biomass (g).

### 2.3. Polyacrylamide Gel Electrophoresis (SDS-PAGE)

The purity of the extracted PE was tested by SDS-PAGE, and its molecular weight was determined. The low molecular weight protein standard (14.4–97.4 kDa) was used as a marker; both the sample and the marker had a sample loading of 10 μL. The electrophoresis condition was with 17 mA constant current and a voltage of 150 V. Coomassie brilliant blue R-250 was used to dye the gel for 15 min after electrophoresis. The gel was decolorized for 8–10 h with decolorizing solution and was finally imaged with protein gel imager.

### 2.4. Optimization of Enzymatic Hydrolysis Conditions

The PE was hydrolyzed by an enzymatic method. Taking the degree of hydrolysis and the degree of color degradation as indicators, the effects of reaction time, reaction temperature, and enzyme dosage on the enzymatic hydrolysis were investigated, respectively. The dissolved PE solution (1 µM, 2 mL) was mixed with enzyme solution with different volumes, and the final enzyme activities were 2000, 5000, 8000, and 11,000 U/g protein, respectively. The mixture was incubated at pH 5.0 (the optimal pH for bromelain activity) in distilled water in a shaker incubator at 200 r/min for 10, 20, 30, and 40 min at 27, 37, 47, and 57 °C, respectively. All incubation mixtures had the same volume at the end of the reaction. The reaction was stopped by inactivating the enzyme by adjusting the pH to the isoelectric point of bromelain at 8.44 with the same volume of NaOH (1 M) to inactivate the enzyme [15]. The samples were centrifuged at 4000 r/min for 15 min to separate enzymes and impurities in the resulting hydrolysates, and the supernatant (protein hydrolysates) was collected. Then, the ultrafiltration centrifuge tubes (3 kDa and 10 kDa) were used to centrifuge the sample at 10,000 r/min for 15 min to obtain a polypeptide (3–10 kDa). The DH and degree of color degradation were used as indicators to measure the hydrolysis extent, as shown in Section 2.4 and Section 2.5. The control sample PE was also conducted by the above hydrolysis procedure but without the addition of bromelain.

### 2.5. Measurement of Degree of Hydrolysis (DH)

The DH of PE was detected using the ortho-phthalaldehyde (OPA) method. The DH was calculated by measuring the decrease in absorbance corresponding to the number of substituted free amino groups, according to the method as previously described [16]. OPA reagent solution (100 mL) was prepared by mixing OPA (80 mg), *β*-mercaptoethanol (200 µL), 10% SDS (*w*/*v*) (5 mL), 92.8 mL sodium tetraborate (0.1 M), and absolute ethanol (2 mL). Two hundred microliters of PEH obtained in selected time points was mixed with OPA reagent (4 mL) at room temperature, and after 2 min at 340 nm, it was measured. The entire hydrolysis of PE was conducted with 6 M NaOH for 6 h at 115 °C [17]. The A_340nm_ was measured, and the DH was calculated using Formula (2):

DH (%) = ((A_t_ − A_t0_)/A_T_) × 100% (2)

where A_t_ was the released amount of free amino group at the selected time point, A_t0_ was the amount of free amino group at 0 h, and A_T_ was the total free amino groups content after complete hydrolysis using 6 M NaOH.

### 2.6. Detection of Color Degradation Rate

The color degradation rate of PE was measured using a UV/Vis spectrophotometer (Agilent Technology, Santa Clara, CA, USA) with a 1 cm pathlength quartz cell. The hydrolysates were diluted four-fold with the distilled water before detecting absorbance within 250–700 nm. The degradation rate of the color was calculated according to Formula (3):

Degradation rate of color (%) = ((A_C_ − A_S_)/A_C_) × 100% (3)

where A_C_ was the absorbance of PE at 565 nm, without the addition of an enzyme during incubation of 1 h at selected conditions of reaction, while AS was the absorbance of PE at 565 nm after treatment, with an enzyme during incubation of 1 h at selected conditions of reaction.

### 2.7. Characterization of PE and PEH

The FTIR spectra of PE and PEH were analyzed by IS50 Fourier transform infrared-spectroscopy (Nicolet, ThermoFisher Scientific, Waltham, MA, USA). The solid-state infrared spectra of PE and PEH in 4000–400 cm^−1^ were recorded by the KBr disk method with a resolution set to 4 cm^−1^. The PE and PEH were, respectively, lyophilized to powders and mixed with KBr (1:100, *w*/*w*) prior to the assay; then, they were ground by an agate mortar and pestle and compressed into granules. The scanning was baseline adjusted with OMNIC 8.2, and the range 1600–1700 cm^−1^ was performed by Fourier auto deconvolution analysis [18].

### 2.8. Analysis of Spectroscopic Properties

UV/Vis scan spectra of PE and PEH were analyzed from the 200–800 nm range using an Agilent 8453 UV spectrophotometer (Agilent Technology, USA). The protein concentrations in the sample were 1 μM, and the working temperature was 25 °C. The PE and PEH solutions were freshly prepared each time before the experiment to minimize experimental error; all spectra were recorded with an incubation time of 5–10 s.

A fluorescence spectrophotometer (Agilent Technology, USA) was used to measure fluorescence spectra of PE and PEH. The scanning emission spectrum was collected in the 300–600 nm range when the exciting wavelength was 290 nm and the excited and emission cracks were 10 nm. The emission spectrums in the range of 500–700 nm were collected when the exciting wavelength was set to 498 nm, and both excited and emission cracks were set to 5 nm. The scanned speed was 240 nm/min. All spectra were recorded after stabilization by incubation for 5–10 s.

### 2.9. Scanning Electron Microscope (SEM)

The morphology of PE and PEH was imaged by SEM (SU-1510, Hitachi, Tokyo, Japan). The conditions were set at 1000× magnification and 20 kV acceleration voltage. Before determination, the double-sided carbon band was on the metal film surface, and the samples were sputtered with gold powder before imaging.

### 2.10. Antioxidant Capacity Analysis

#### 2.10.1. Radical Scavenging Ability of PE and PEH—DPPH Assay

DPPH radical scavenging activity was determined according to a reported method and was adapted to a 96-well flat bottom plate [19]. Each sample was mixed in a tube of 1 mL and transferred to the well. The samples (0.5 mL) were mixed with 0.5 mL of 0.2 mM DPPH radical solution dissolved in 80% ethanol. The absorbance at 517 nm was detected after reacting for 30 min. As the control, ethanol was used instead of the sample to measure the absorbance, and in the blank group, the absorbance was measured by ethanol. The antioxidant activities of the samples were assessed by the percent inhibition of DPPH using Formula (4):

DPPH radical scavenging activity (%) = (1 − (Ds − D_b_)/D_c_) × 100% (4)

where D_s_, D_c_, and D_b_ were the absorbance of test sample, the control group, and the blank, respectively.

#### 2.10.2. Radical Scavenging Ability of PE and PEH—ABTS Assay

The ABTS free radical scavenging activity of PE and PEH was determined by an ABTS free radical scavenging ability detection kit, and the operation followed the instructions of the kit (BC4770-50T/24S). The sample (50 μL) was mixed with buffer four working solution (100 μL) and ABTS working solution (850 μL). The mixtures were mixed well in the Eppendorf (EP) tube, and were standing for 6 min in the dark, followed by detection at 405 nm. The antioxidant components contained in the sample solution can react with the ABTS+ solution to cause the reaction system to fade, resulting in a decrease in the absorbance at 405 nm. The control group was defined as the absorbance of the tube without the addition of a sample, and the sample group was defined as the absorbance of the tube with a sample and ABTS+. The scavenging activity was calculated according to Formula (5):

ABTS radical scavenging activity (%) = ((A_0_ − (A_S_ − A_C_))/A_0_) × 100% (5)

where A_0_ was the absorbance of the control without sample, As was the absorbance in the presence of the sample and ABTS+, and Ac was the absorbance of the sample blank without ABTS+.

### 2.11. Effect of Temperature, Light, and Metal Ion on the Stability of PEH

To study the effect of temperature, visible, and UV light on the stability of PEB in the form of PE and PEH, the changes of the absorbance at 565 nm were detected using a UV/Vis spectrometer. The PE was used as the control sample and was performed in the same procedure for the PEH. Each sample (2 mL) was diluted to 0.24 mg/mL and was heated for 30 min at 30, 40, 50, 60, 70, and 80 °C, respectively. The color change of the solution was observed by detecting A_565_. The change in relative absorbance was evaluated as the ratio of A_565_ at the start of thermal treatment (A_25°C_) to A_565_ after a certain heat treatment. The experiments were carried out in three parallels, the experiments were carried out in the dark, and their changing trends were recorded.

The stability of PEB in the form of PE and PEH was studied by detecting the changes of the absorbance peak at 565 nm, which is expressed as the ratio of A_565_ at the beginning of light treatment to A_565_ after certain light treatment. A total of 2 mL of PE and PEH solutions were taken, respectively, and diluted to 0.24 mg/mL. The solution was incubated successively under visible and ultraviolet light at room temperature for different times to observe the color change of the solution, and the absorption value was scanned in 400–700 nm. The visible light source was a fluorescent lamp, and each sample was incubated successively for 2, 4, 6, and 8 h at room temperature. The UV light source was a UV bactericidal lamp (254 nm), and each sample was incubated successively for 0.5, 1.0, 1.5, 2.0, 2.5, and 3 h at room temperature. The experiments were carried out in three parallels.

The influence of different metal ions, including Fe^3+^, Cu^2+^, Mn^2+^, Mg^2+^, and Zn^2+^(the final concentrations were 1 mM), on the stability of PEB in PE and PEH was conducted by mixing the metal ions with the PE and PEH (0.24 mg/mL) solution. The absorbance value at 565 nm was measured immediately after incubation at 4 °C for 10 min in darkness. The stability of PEB in the form of PE and PEH was calculated by detecting the changes of the absorbance peak at 565 nm, which was expressed as the ratio of A_565_ before the metal ions treatment to A_565_ after metal ions treatment. The experiments were carried out in three parallels.

### 2.12. Measurement of Color

The trichromatic color coordinates of PE and PEH was studied by an instrument colorimeter (CS-810, Huangzhou, China) with a cuvette. The sample was shaken well before measurement and was calibrated with standard blackboard and standard whiteboard. The spectrophotometer was calibrated prior to the analysis, and the ΔE was fitted by Formula (6) [20]:

ΔE = ((L_n_ − L_m_)^2^ + (a_n_ − a_m_)^2^ + (b_n_ − b_m_)^2^)^1/2^ (6)

where L represented light/dark, a and b were expressed as red/green and yellow/blue, respectively, and n and m represented the final and initial storage times, respectively.

### 2.13. Statistical Analysis

Statistical analyses were performed using the statistical software package for the social sciences (SPSS, software version 17.0, IBM SPSS, Chicago, IL, USA) through one-way ANOVA and Duncan’s multiple range test, with a significance level of *p* < 0.05.

## 3. Results and Discussion

### 3.1. Preparation of PE and PEH

Figure 1b depicts the flow chart of the preparation of PE and PEH. The extraction process of R-type PE was carried out at a low temperature in a dark environment. It was prepared by freeze-thaw, crushing, homogenization centrifugation, and salting out. The yield of PE was 0.2% (*w*/*w*). Optimal conditions for hydrolysis of PE by bromelain was 8000 U/g during 30 min at 47 °C. PEH was obtained after the inactivation of the enzyme at its pI and by removing the pellet by centrifugation. The resulting supernatant was further filtered by ultrafiltration using a molecular weight cut-off of 3–10 kDa. The PE was analyzed by SDS-PAGE (Figure 1c). The relative molecular masses of the *α*, *β*, and *γ* subunits were about 19, 21, and 27 kDa, respectively [3]. The electrophoresis results did not show the specific bands corresponding to the PEH subunits, mainly because bromelain degraded the PE and the ultrafiltration (3–10 kDa) led to a number of peptides below 10 kDa.

### 3.2. Optimization of Enzymatic Hydrolysis Conditions of PE by Bromelain

The DH is a key index for tracking and controlling proteolytic reactions and is a parameter for the length of broken peptide chains and peptide bonds in macromolecules [21]. The use of DH for the indication of degradation rate of PE is accepted mainly because DH represents the changes in the free amino group, as shown in Formula (2). An increase in DH indicates an increase content of the free amino acids; thus, the DH can be used as an index for the representation of protein degradation. In addition, it is related to peptide size or structure, which affects exposure and biological activity of certain amino acids. PE is a kind of natural pigment protein. As mentioned in the introduction section of the article, PE is a relatively unstable protein that is easily affected by temperature and light. Enzymatic hydrolysis usually affects the stability of proteins by changing the reaction conditions, such as reaction time, temperature, and enzyme dosage, leading to the degradation of protein into peptides and color loss, which limits the application range of PE to a certain extent.

#### 3.2.1. The Effect of Reaction Time on Enzymatic Hydrolysis

**Figure 2:**
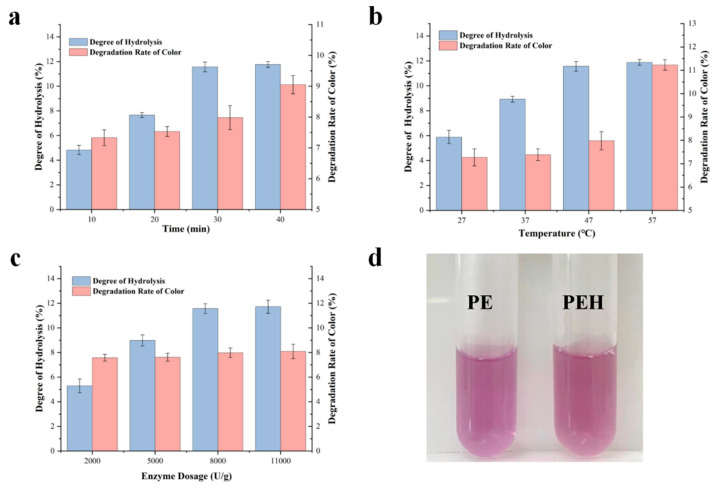
(**a**) Effects of reaction time on hydrolysis rate and color degradation rate (*n* = 3) of PE. (The reaction temperature was 47 °C and the enzyme dosage was 8000 U/g). (**b**) Effects of reaction temperature on hydrolysis rate and color degradation rate (*n* = 3) of PE. (The reaction time was 30 min and the enzyme dosage was 8000 U/g). (**c**) Effects of enzyme dosage on hydrolysis rate and color degradation rate (*n* = 3) of PE. (The reaction temperature was 47 °C and the reaction time was 30 min). (**d**) Photographs of PE and PEH before (**left**) and after (**right**) enzymatic hydrolysis.

In the initial stage of the hydrolysis reaction, the diffusion rate of PE to the active center of bromelain accelerated with time duration, and the DH reached the highest at 40 min (Figure 2a). The lower concentration of soluble peptides may compete less with a substrate, leading to a rapid increase in DH from 20 to 30 min. With a further extension of the reaction time to 40 min, the prolonged incubation period will not continue to increase the hydrolysis (11.76 ± 0.23%), and the DH remains stable with no significant changes relative to that of 30 min (*p* > 0.05), indicating that the hydrolysis process may be drooped due to lower susceptibility to protease hydrolysis and the generation of the peptides [22]. Moreover, the color loss extent also exhibited an increasing trend, and it can be found that the color degradation rate at the reaction time of 40 min was 9.05 ± 0.30%, which is significantly higher than that for the reaction at 10, 20, and 30 min (*p* < 0.05). Therefore, the optimal reaction time of 30 min was chosen for further preparation of PEH.

#### 3.2.2. Influence of Reaction Temperature on Enzymatic Hydrolysis

Temperature can significantly influence the hydrolysis of protease. When the reaction time was 30 min and the enzyme dosage was 8000 U/g, as the treated temperature increased from 27 °C to 57 °C, the DH increased significantly (*p* < 0.05) below 47 °C and the degradation value reached the highest value of 11.88 ± 0.23% at 57 °C (Figure 2b). A high temperature greater than 57 °C was not conducted, and the optimum temperature for the bromelain is around 55 °C [23]. A high temperature may inactivate the enzyme, owing to the unfolding of its protein structure and thermal denaturation [24]. There were no significant differences for the color degradation rates when the temperature was ranging from 27 to 47 °C (*p* > 0.05), and the color degradation rate reached 11.23 ± 0.22% at 57 °C, indicating that the color of PEH was seriously damaged under high temperature conditions. This finding was consistent with the previous report that the light absorption value of PEB will be significantly reduced under high temperature conditions due to the loss of pigment. Therefore, the treatment at 47 °C was chosen to balance the hydrolysis degree and the color loss of the PEH.

#### 3.2.3. The Effect of Enzyme Dosage on Enzymatic Hydrolysis

With the increase of the enzyme amount from 2000 to 8000 U/g, the DH of phycoerythrin increased and showed no significant increase when the enzyme amount was 11,000 U/g (Figure 2c). The increased DH value resulted from the increased presence of amino acids and smaller peptides in the hydrolysates. As the enzyme concentration increases, more active sites on the enzyme become available, resulting in significant cleavage of peptide bonds and massive protein solubilization. However, as the amount of enzyme increased to a certain extent, such as 11,000 U/g, the contact of enzyme with the substrate molecule PE decreased, eventually reducing the DH to 11.72 ± 0.53%, reaching a stable stage where no significant hydrolysis occurred. This situation may also be related to product inhibition due to substrate supersaturation or auto-digestion of the enzyme [25]. In addition, Figure 2c showed that the degradation rate of color of PEH remained unchanged with no significant difference in the color degradation rates (*p* > 0.05) by increasing the enzyme concentration, and the 8000 U/g dose was chosen to retain the phycoerythrin color and, meanwhile, maximize the DH.

In sum, the PEH was prepared by enzymatic hydrolysis of PE with bromelain (8000 U/g) at 47 °C for 30 min, with a degree of hydrolysis (DH) of 11.57 ± 0.39% and a color degradation rate of 7.98 ± 0.39%. Hydrolysis of PE by bromelain occurred according to the Michaelis–Menten kinetics. According to the obtained results, after 30 min of reaction, the substrate was completely hydrolyzed. At this condition, the PEH was imaged and showed that the typical purple color was maintained and was deeper than the pure PE sample (Figure 2d), indicating that pigment was concentrated in the PEH with a molecular weight of 3–10 kDa.

### 3.3. UV/Vis Absorption and Fluorescence Spectroscopy of PE and PEH

**Figure 3:**
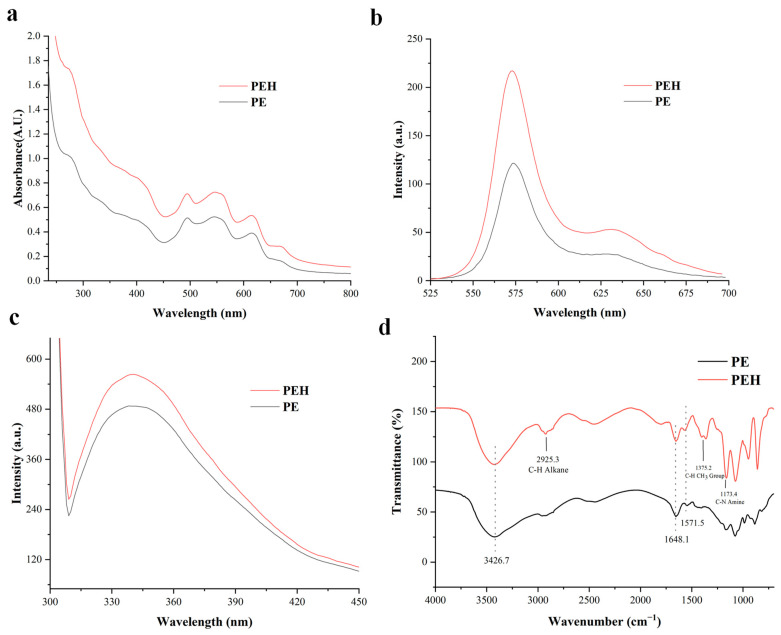
(**a**) UV/Visible, (**b**) fluorescence (Ex 498 nm), and (**c**) (Ex 290 nm) spectra of PE and PEH (1 μM). (**d**) FTIR spectra of PE and PEH.

The conformational changes caused by enzymatic hydrolysis may lead to a change in resonance energy transfer efficiency, which is reflected in the spectral properties of the protein. Figure 3a shows that the UV/Vis absorption spectrum of PE had a peak at 565 nm, a characteristic absorption peak of phycobilin in PE attributed to PEB. Because the concentration of PE and PEH was the same (1 µM), the increase in the intensity of the characteristic peaks corresponding to PEB is speculated to be due to the enrichment of more pigment molecules, and the enrichment phenomenon may be related to the generation of peptides and changes of the spatial array of the microenvironment around PEB [26].

Because of the binding of (*αβ*)_6_*γ* to phycobilin chromophores, PE can highly produce fluorescence, and its absorption coefficient and quantum yield are better than other fluorescent proteins. Figure 3c shows that when the excitation wavelength is 290 nm, PE has a fluorescence absorption peak near 340 nm, which is mainly due to the tryptophan group in the protein. The increase by 10.87% of fluorescence intensity after enzymatic hydrolysis proved the effectiveness of enzymatic hydrolysis [27]. Figure 3b shows that the fluorescence absorption peak at 575 nm excited at 498 nm is related to the pigment domain. The fluorescence intensity increased by 13.9% after enzymatic hydrolysis but without peak shift. This process caused the enhancement of fluorescence intensity, which was inferred to be related to the changes of PEB concentration and changes in the binding behavior to (*αβ*)_6_ [14]. It showed that the pigment can be concentrated in the molecular range of 3–10 kDa after enzymatic hydrolysis. Due to the structural stability of PE oligomeric state (*αβ*)_6_, it can still maintain its fluorescence characteristics when the external environment changes. Therefore, when the structure of PE was changed due to enzymatic hydrolysis, the fluorescence intensity increased, and the spectral sensitivity of R-PE and its enzymatic degradation products can be clearly observed.

### 3.4. FTIR Analysis of PE and PEH

Figure 3d shows that the characteristic protein bands of PE and PEH were at 3426.7, 1648.1, and 1571.5, respectively. These three bands represent the O−H stretching vibration of amide A band [28], the stretching vibration of C = O in the polypeptide chain, and the stretching vibration of the C-N bond and the bending vibration of the N-H bond [29], indicating that the *α*-helix is the main component of the secondary structure of PE and PEH. Specifically, PEH showed more functional groups in the FTIR spectrum relative to PE, such as the C-H symmetrical bending vibration of methyl group (1375.2 cm^−1^), alkane C-H stretching of amines (2925.3 cm^−1^), and C-N stretching vibrations of amines (1173.4 cm^−l^) [30]. These differences proved that the secondary structure of the mixture was changed after bromelain treatment due to the unfolding of the protein structure or the cleavage of the protein into peptides.

Based on the FTIR spectrum, the contents of the secondary structure of these samples were calculated and are shown in Table 1. The increased *α*-helix content and the decreased *β*-sheet and random coil contents in PEH suggested that the PE structure was susceptible to enzymatic hydrolysis [31]. An increase in the number of *α*-helix structures can improve the protein stability, owing to the formations of rigid *α*-helix coils. Moreover, the number of *α*-helix increased, which can facilitate pigment stability [32]. Therefore, the enzymatic hydrolysates of PE would be different in physicochemical and functional properties relative to PE.

### 3.5. SEM Morphology

**Figure 4:**
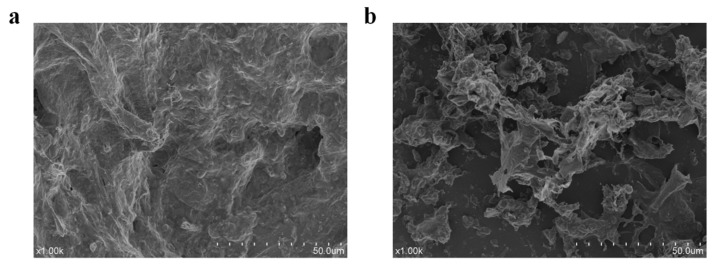
SEM images of (**a**) PE and (**b**) PEH at 1000 magnification.

To better understand the structural effects of bromelain hydrolysis on phycoerythrin, scanning electron microscopy images of the samples were taken before and after the enzymatic hydrolysis process. Scanning electron microscopy clearly showed that the enzymatic hydrolysis of PE affected its microstructure (Figure 4). PE had a relatively complete structure, compact texture, and smooth appearance (Figure 4a), but the PEH after enzymatic hydrolysis by bromelain had a large number of stomata-like broken microstructures, the internal structure was loose, and its surface had obvious residual prismatic skeleton structure after degradation (Figure 4b). The changes of microstructure after enzymatic hydrolysis indicated that the enzymatic hydrolysates increased the exposure degree and exposed area of the active site of biomass and reduced the structural integrity of protein molecules. These structural changes may influence the binding of pigment molecules with the protein/peptide and have a significant impact on the improvement of biological activity [33].

### 3.6. Antioxidant Capacity

**Figure 5:**
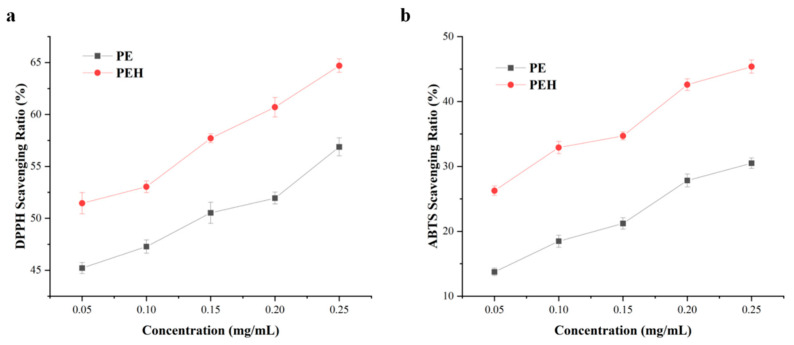
Effects of PE and PEH on scavenging free radicals of (**a**) DPPH and (**b**) ABTS. Values are means ± SD (*n* = 3).

The entities that can inhibit or slow down the oxidative damage caused by free radicals are considered antioxidants [34]. The antioxidant activities, including the DPPH and ABTS free radical scavenging of PE and PEH, were explored (Figure 5), and a concentration-dependent trend was found for these two samples. Results indicated that the antioxidant activity of PEH was significantly higher than that of PE, the ability of scavenging DPPH free radicals was increased by an average of 7.15% (*p* < 0.05), and the ability of scavenging ABTS free radicals increased by an average of 14.02% (*p* < 0.05). The scavenging rates of ABTS and DPPH free radicals of active polypeptides all increased with the increase of the concentration of substrate. This indicates that hydrolysis alters the distribution of amino acids, and it is inferred that the amino acid residues and the new peptide molecules in the PEH have strong antioxidant capacities [35]. By analyzing the experimental results of spectroscopy, we speculated that the change of pigment environment may also be a reason for the improvement of antioxidant activity. These results demonstrate that commercial bromelain has the potential for enzymatic hydrolysis of PE and the improvement of PE value for its utilization in functional foods.

### 3.7. Color Stability against Environmental Conditions

**Figure 6:**
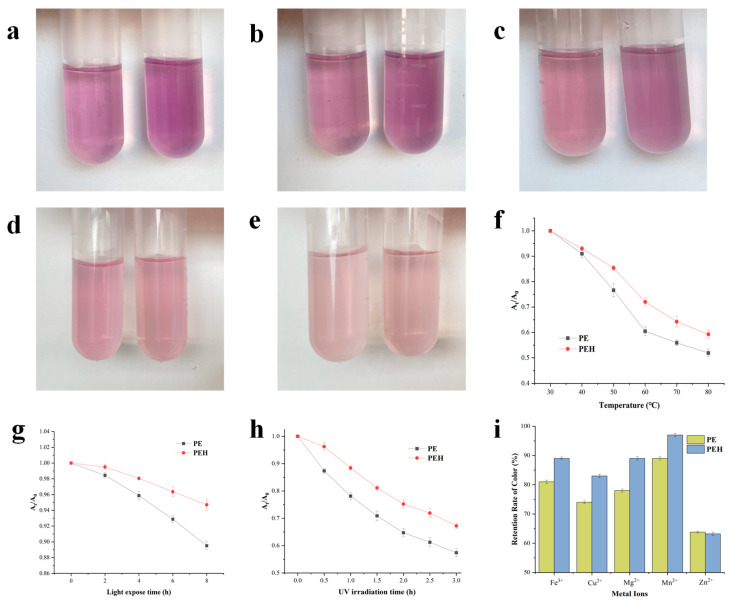
Photographs of PE and PEH heated to (**a**) 40 °C, (**b**) 50 °C, (**c**) 60 °C, (**d**) 70 °C, and (**e**) 80 °C for 30 min under the condition of avoiding light (left: PE, right: PEH). (**f**) Change of relative absorbance At/A_0_ (λ = 565 nm) of PE and PEH after thermal treatment. (**g**) Change of relative absorbance At/A_0_ (λ = 565 nm) of PE and PEH after natural light irradiation treatment. (**h**) Change of relative absorbance At/A_0_ (λ = 565 nm) of PE and PEH after ultraviolet light irradiation treatment. (**i**) Change of relative absorbance At/A_0_ (λ = 565 nm) of PE and PEH after metal ions treatment. Values are means ± SD (*n* = 3).

The PEB of PE usually absorbs strongly at the 480–570 nm regions, and heat-induced degradation of PE was studied by detecting the absorption peak at 565 nm to analyze the stability change of the pigment before and after enzymatic hydrolysis. Figure 6a–e presents the photos taken of the PE and PEH samples heated to 40 °C, 50 °C, 60 °C, 70 °C, and 80 °C for 30 min under the condition of avoiding light at pH 6.8. The state of PE and PEH was basically the same at 40 °C. As the temperature gradually increased, it was observed that the fading degree of PE was significantly higher than that of PEH (*p* < 0.05), and PEH retained a deeper color and showed higher temperature stability. The relative changes in absorbance were evaluated by the ratio of A_565_ after a specific heat treatment (A_t_) to A_565_ in the beginning of the heat treatment (A_0_) (Figure 6f). The result showed that the stability of PE and PEH decreased with the temperature from 30 to 80 °C. Experiments showed that enzymatic hydrolysis by bromelain could enhance the stability of pigment. For example, after 60 °C treatment, PEH retained more than 10.0% of A_t_/A_25°C_ over PE. Furthermore, the solution color gradually became lighter, demonstrating that they all lost color to different extents, while the loss of PEH was relatively lower. Thus, the enzymatic hydrolysis by bromelain enhanced the thermal stability of the PEB. The changes in the arrangement and distribution of the PEB with the neighboring amino acids in PEH may improve the resistance of PEB to thermal treatment and attribute to enhanced stability [36].

The light stability of PE and PEH was assessed by measuring the absorption attenuation after natural light exposure. The changes in relative absorbance were analyzed as the ratio of A_565_ after specific light exposure treatments (A_t_) to the A_565_ in the start of the treatments (A_0_) (Figure 6g). The results showed that the A_t_/A_0_ values gradually decreased for all samples (2 to 8 h). For example, the A_t_/A_0_ of PE attenuated by 10.49%, while the At/A_0_ of the PEH only decreased by 5.3% from 2 to 8 h, suggesting that bromelain can improve light stability by slowing the denaturation rate of PE. In contrast, UV light resulted in a more significant reduction in A_t_/A_0_ for PE and PEH (*p* < 0.05) (Figure 6h). The common point was that PEH retains more A_t_/A_0_ than PE. Thus, the hydrolysis of PE by bromelain also enhanced the UV exposing stability of PEB. The improved stability of PEH is attributed to the binding of chromophores to peptides released in the hydrolysates, thus protecting the rigid conformation of phycoglobin from damage. The chromopeptides generated from hydrolysis may contain more amino acid residues that can interact with chromophores [26], which is beneficial for the stabilization of the chromophores.

In the process of food production, storage, and transportation, food can be exposed to various metals. The combination of metal ions with PE and PEH may affect their ultraviolet absorption and stability. It can be seen from Figure 6i that with the addition of different metal ions (Fe^3+^, Cu^2+^, Mn^2+^, Mg^2+^ and Zn^2+^), PEH showed a higher stability than PE against all these metal ions (*p* < 0.05) except Zn^2+^. Where Cu^2+^ had the strongest effect on the attenuation of the pigment, the A_t_/A_0_ of the PEH increased by 9.0% to that of PE. Mn^2+^ showed a relatively lower effect on the stability. Zn^2+^ showed little effect on its stability (*p* > 0.05). These results indicate that the enzymatic hydrolysis of bromelain could effectively protect the color against metal ions. We speculated that the metal ions may interact with the PEB with conjugated double bonds in PEH in a different manner with that of PE, leading to the difference in the intensity of the absorption wavelength [37].

### 3.8. Aberration Analysis of PE and PEH

**Figure 7:**
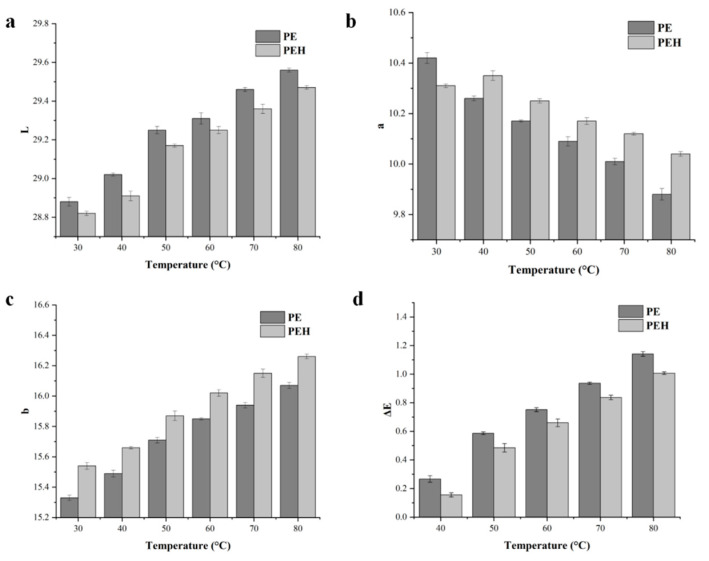
Effect of thermal treatment on the color change of PE and PEH. (**a**) L-value change. (**b**) a-value change. (**c**) b-value change. (**d**) ΔE value change. Values are means ± SD (*n* = 3).

To further study the color changes of the sample, L-, a-, and b-values of PEH were tested using a chromatometer, and PE served as a control sample. Positive L-values usually describe the brightness of the sample. Brightness variation (ΔL) of PE is generally much higher than PEH because PE is degraded faster and tends to be more transparent (Figure 7a). For example, after 70 °C treatment, the ΔL in PE was 0.58, which was significantly higher than that in PEH (*p* < 0.05). Figure 7b showed that the changes in the a-values in both samples were decreased, indicating that the red color of all samples decreased after heat treatment, but this value was less reduced than the L- and b-values. Specifically, the PEH sample decayed less than the PE sample after the same temperature treatment. The changes in b-values are shown in Figure 7c. The increases in the b-values were observed in two of these samples, and the PEH samples showed higher b-values. Another important parameter is ΔE, which represents the overall color difference, as shown in Figure 7d. After heat treatment, the calculated (ΔE) in PE was much higher than the calculated value in PEH (*p* < 0.05), indicating that the overall color change in PEH samples was much lower than in PE. Furthermore, the higher the temperature, the greater the difference in ΔE between the PE and PEH samples, which proved that the PEH could bear higher thermal treatments. We inferred that the color protection of the PEH after enzymatic treatment resulted from non-covalent interaction between the peptide and chromophore [26].

The color changes against natural light and ultraviolet light irradiation were performed to analyze the color stability of PEH. Similar results were found in Figures S1 and S2, Supporting Information, and demonstrated that the enzymatic hydrolysis with bromelain could be used as a method to improve the stability of pigment against natural/ultraviolet light irradiation-induced fading of PE. This result reminded us of a different study about the stabilization of PE by the binding of oligochitosan molecules [38]. This reported binding method was absolutely different from the enzymatic means in our study, and these two methods were both effective. Therefore, enzymatic hydrolysis can serve as a new reference for the protection of color stability of PE. At present, the exact mechanism about the structure and activity of PEH in nature is uncertain, additional studies in greater detail are required, and we expect a larger correlation between the chromophore and peptide structures and relevant functions in future work.

The traditional method usually uses the introduction of another substance to stabilize PEB, such as using biotinylated antibiotics and chitosan oligosaccharides to bind to PE [38,39]. The method of using bromelain to hydrolyze PE introduced in this article is feasible because the enzymatic hydrolysis method has the advantages of being mild, safe, and environmentally friendly, and the process of stabilization does not require the compounding of other substances. Compared to traditional stabilization methods, this is a new exploration, and the effect is quite significant.

## 4. Conclusions

In order to improve the degradation and denaturation of natural pigments, we used bromelain enzymatic hydrolysis to treat PE, analyzed and compared the structural changes before and after enzymatic hydrolysis, and made reasonable evaluation on the improvement of properties. In this work, PEH was prepared by enzymatic hydrolysis of PE with bromelain. The optimal condition was obtained with a 0.5 h, 47 °C, and 8000 U/g reaction. The enzymatic hydrolysis of PE by bromelain can alleviate the fading degree of PE under high temperatures, light (natural and ultraviolet light), and metal ion exposure, thus improving the color stability of PE. Meanwhile, the antioxidant capacity of PEH increased with the increase of the concentration of the substrate. Compared with traditional methods by embedding technology or applying high concentration of sugars to stabilize the PE, this scheme shows superiorities in its simple operation procedure and a low usage amount of the bromelain. This finding can provide an effective scheme for preservation and function enhancement of the protein–pigment complex and offers a new approach to extend the applications of PE as a natural color additive in the food industry, such as in bubble gum, beverages, milky products, and jelly, as well as the chemical analysis, such as fluorescent labelling uses.

## Figures and Tables

**Table 1:** Content of the secondary structure of PE and PEH.

Sample	*β*-Sheet	Random	*α*-Helix	Turn	*β*-Anti
PE	44.8 ± 0.32	16.12 ± 0.36	7.18 ± 0.21	29.65 ± 0.57	2.25 ± 0.28
PEH	35.4 ± 0.45	7.63 ± 0.33	16.33 ± 0.46	33.27 ± 0.31	7.37 ± 0.42

Results are expressed as mean value ± SD (*n* = 3).

## Data Availability

The data used to support the findings of this study can be made available by the corresponding author upon request.

## Supplementary Materials

The following supporting information can be downloaded at: https://www.mdpi.com/article/10.3390/foods12132574/s1, Figure S1. Effect of natural light irradiation treatment on the color change of PE and PEH; Figure S2. Effect of ultraviolet light irradiation treatment on the color change of PE and PEH.
