# Supplementary material for: The Hydrolysis of Pigment-Protein Phycoerythrin by Bromelain Enhances the Color Stability
Source: Foods. 2023 Jun 30;12(13):2574. doi: 10.3390/foods12132574 (PMC10340757; doi:10.3390/foods12132574)
Supplement: Supplementary file 1 [file foods-12-02574-s001.zip › foods-2436045-supplementary.pdf]

The hydrolysis of pigment-protein phycoerythrin by bromelain enhances the color stability

Yifei Sun,[a,1] Yuanmeng Cui,[a,1] Ruhua, Wang,[a] Junrui Ma,[a] Haili Sun,[a] Lei Cheng,[b,*] Rui Yang,[a,*]

[a] State Key Laboratory of Food Nutrition and Safety, College of Food Science and Engineering, Tianjin University of Science & Technology, Tianjin, 300457, China.

[b]Beijing Engineering and Technology Research Center of Food Additives, Beijing Technology & Business University (BTBU), Beijing, China.

[1]These authors contributed equally to this work.

*Corresponding authors

Dr. Rui Yang (yangrui@tust.edu.cn) and Dr. Lei Cheng (chenglei@btbu.edu.cn）

Tianjin University of Science & Technology

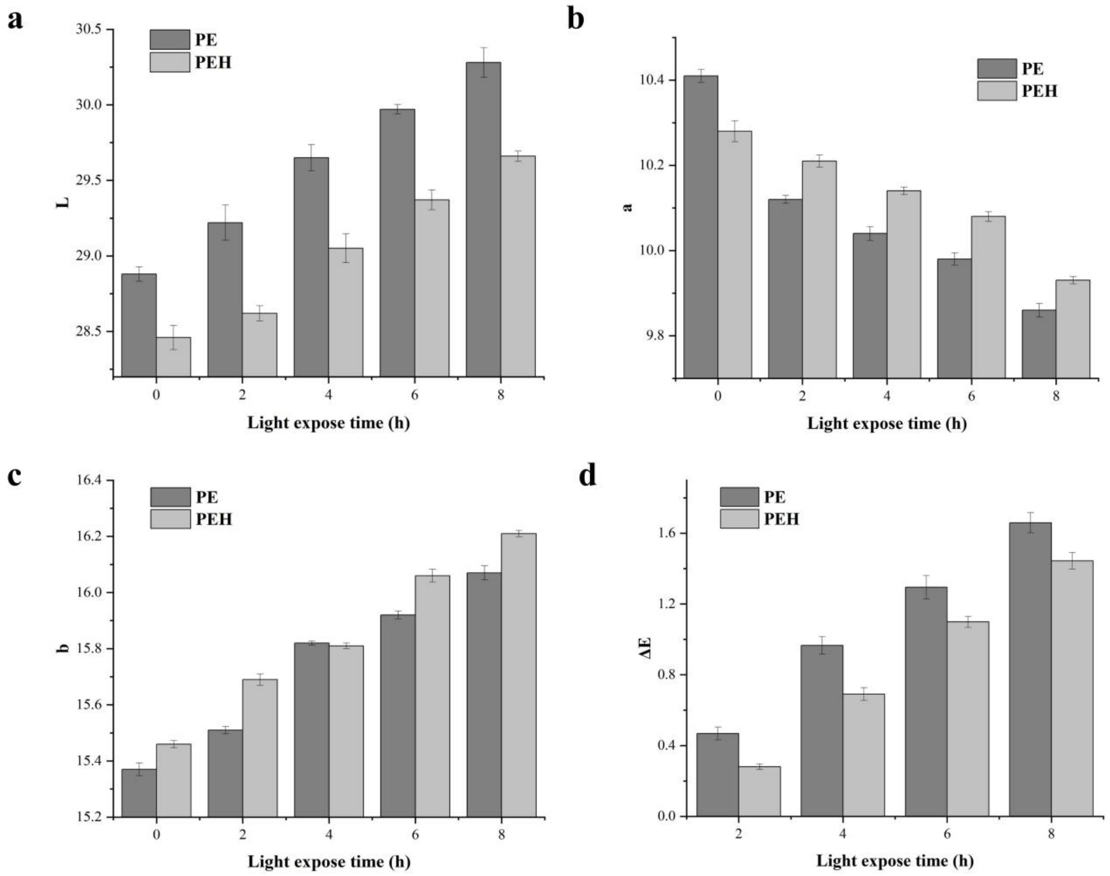

Figure S1. Effect of natural light irradiation treatment on the color change of PE and PEH. (a) L value change. (b) a value change. (c) b value change. (d) ΔE value change. Values are means ± SD (n = 3).

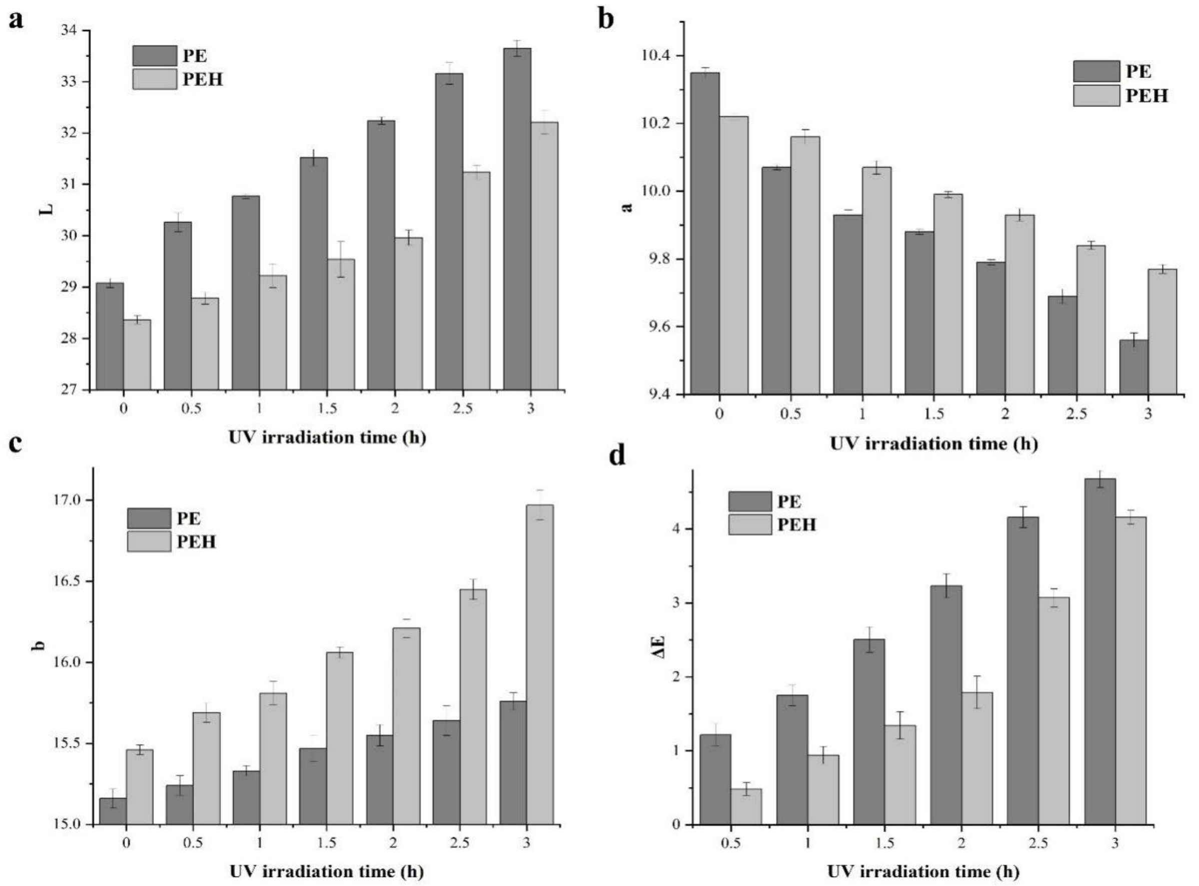

Figure S2. Effect of ultraviolet light irradiation treatment on the color change of PE and PEH. (a) L value change. (b) a value change. (c) b value change. (d) ΔE value change. Values are means ± SD (n = 3).
